# Trends in 30-day readmissions following hospitalisation for heart failure by sex, socioeconomic status and ethnicity

**DOI:** 10.1016/j.eclinm.2021.101008

**Published:** 2021-07-14

**Authors:** C Lawson, H Crothers, S Remsing, I Squire, F Zaccardi, M Davies, L Bernhardt, K Reeves, R Lilford, K Khunti

**Affiliations:** a Department of Cardiovascular Sciences, University of Leicester, and NIHR Cardiovascular Biomedical Research Centre, Glenfield Hospital, Leicester, UK; b Real World Evidence Unit, University of Leicester, UK; c University of Birmingham, UK; d Diabetes Centre, University of Leicester, UK

**Keywords:** Heart failure, hospitalisation, Readmission, AF, Atrial fibrillation, CI, Confidence Interval, COPD, Chronic obstructive pulmonary disease, CRT, Cardiac resynchronisation therapy, CVA, Cerebrovascular accident, CVD, Cardiovascular disease, HES, Hospital Episode Statistics, HF, Heart failure, ICD, Implantable cardioverter defibrillator, IHD, Ischaemic heart disease, IMD, Index of Multiple Deprivation, ONS, Office of National Statistics, MI, Myocardial infarction, PCI, Percutaneous coronary intervention

## Abstract

**Background:**

Reducing the high patient and economic burden of early readmissions after hospitalisation for heart failure (HF) has become a health policy priority of recent years.

**Methods:**

An observational study linking Hospital Episode Statistics to socioeconomic and death data in England (2002-2018). All first hospitalisations with a primary discharge code for HF were identified. Quasi-poisson models were used to investigate trends in 30-day readmissions by age, sex, socioeconomic status and ethnicity.

**Findings:**

There were 698,983 HF admissions, median age 81 years [IQR 14].

In-hospital deaths reduced by 0.7% per annum (pa), whilst additional deaths at 30-days remained stable at 5%. Age adjusted 30-day readmissions (21% overall), increased by 1.4% pa (95% CI 1.3-1.5). Readmissions for HF (6%) and ‘other cardiovascular disease (CVD)’ (3%) remained stable, but readmissions for non-CVD causes (12%) increased at a rate of 2.6% (2.4-2.7) pa. Proportions were similar by sex but trends diverged by ethnicity. Black groups experienced an increase in readmissions for HF (1.8% pa, interaction-p 0.03) and South Asian groups had more rapidly increasing readmission rates for non-CVD causes (interaction-p 0.04). Non-CVD readmissions were also more prominent in the least (15%; 15-15) compared to the most affluent group (12%; 12-12). Strongest predictors for HF readmission were Black ethnicity and chronic kidney disease, whilst cardiac procedures were protective. For non-CVD readmissions, strongest predictors were non-CVD comorbidities, whilst cardiologist care was protective.

**Interpretation:**

In HF, despite readmission reduction policies, 30-day readmissions have increased, impacting the least affluent and ethnic minority groups the most.

**Funding:**

NIHR.

## Abbreviations

AF: Atrial fibrillation
CI: Confidence Interval
COPD: Chronic obstructive pulmonary disease
CRT: Cardiac resynchronisation therapy
CVA: Cerebrovascular accident
CVD: Cardiovascular disease
HES: Hospital Episode Statistics
HF: Heart failure
ICD: Implantable cardioverter defibrillator
IHD: Ischaemic heart disease
IMD: Index of Multiple Deprivation
ONS: Office of National Statistics
MI: Myocardial infarction
PCI: Percutaneous coronary intervention

**Research in context**

**Evidence before this study**

Reducing the high patient and economic burden of early readmissions after hospitalisation for heart failure (HF) has become a health policy priority of recent years. Epidemiological reports on readmissions have been conducted mostly at an aggregate level, which does not take account of differences in readmissions over time between subgroups of people with HF, or between specific causes of readmission. There is a key evidence gap on 30-day readmission trend data among different population groups.

**Added value of this study**

By linking national hospital and death data (between 1st January 2002 and 31^st^ December 2018), we were able to report national trends in 30-day readmissions over 16 years, by cause and in different population groups defined by age, sex, ethnicity and socio-economic status. We found that, in HF patients in England, despite readmission reduction policies, 30-day readmissions have increased and this increase was largely explained by non-cardiovascular causes, with the highest rates in South Asian groups and in the least affluent groups. Whilst readmissions for HF were stable, there were diverging trends by ethnicity, with Black groups experiencing a significant increase in readmissions over time.

**Implications of all the available evidence**

Early readmissions in heart failure presents a major and persistent public health challenge. Increasing readmissions for non-cardiovascular causes as well as diverging trends by population groups means that readmission reduction programmes require close consideration of patient factors and a multidisciplinary approach to specialist non-cardiovascular care.

Alt-text: Unlabelled box

### 1 Introduction

Heart failure (HF) is the fastest growing cardiovascular condition worldwide, conferring substantial clinical and economic challenges to health services [1]. Globally, an estimated $108 billion, constituting 2% of the healthcare budget, is spent on HF each year [2]. This figure is predicted to rise over the next two decades [3] and predominantly relates to high hospitalisation rates [4], half of which are thought to be potentially avoidable [5]. Furthermore, admission rates for people with HF are increasing, with group disparities according to sex, socioeconomic status and ethnicity [6].

One of the strongest predictors of hospital admission for patients with HF is a recent prior admission [7]. Around 22% of patients admitted to hospital with HF experience potentially avoidable readmissions soon after discharge, which are associated with high costs and poor prognosis [8]. Consequently, reducing 30-day readmissions has been a longstanding target for governments worldwide, to simultaneously reduce costs and improve quality of care [9]. Yet, in HF, national policy directives to reduce unnecessary readmissions among different countries in Europe and the US, have only shown minimal success over the past twenty years.

In the US, readmission reduction programmes, alongside financial incentives, reduced 30-day readmissions by only 2% over a decade [10], with bigger reductions per annum for primary versus secondary HF admissions [11] and with wide variation according to differences in health services and care provision [12]. In Europe, reports have been conflicting with Spain reporting increases in all readmissions [13] and Sweden reporting reductions in cardiovascular readmissions, alongside increasing non-cardiovascular readmissions [14]. In UK, 18% of patients with HF are readmitted within 30-days [15], but evidence on trends over time are scarce. Furthermore, whilst a few studies have shown variation in readmission rates according to population characteristics such as sex [16], socioeconomic status [17,18], and ethnicity [19], [20], [21], there is a key evidence gap on 30-day readmission trend data among different population groups. This evidence is key for understanding readmission rates in a changing population demographic and for improving readmission reduction by targeting the highest risk groups. The aim of this study was to investigate trends in 30-day readmissions after hospitalisation for HF, by age, sex, socioeconomic status and ethnicity.

### 2 Methods

#### 2.1 Study population

We used the Hospital Episode Statistics (HES) database linked to Office of National Statistics (ONS) death data and Index of Multiple Deprivation (IMD) socioeconomic data. HES contains details of all admissions, outpatient appointments and emergency department attendances at all public hospitals in England. Data includes patient sociodemographic information such as age, sex and ethnicity, administrative data and all diagnoses coded using International Classification of Diseases version 10 codes (ICD-10) and all procedures using OPCS Classification of Interventions and Procedures (OPCS-4). Each discharge has one primary diagnosis and up to 19 secondary diagnoses covering comorbidities and during admission complications. This observational study was registered with the local Clinical Audit Department (Clinical Audit Registration and Management System number 14626) and did not require ethics approval or patient consent. Data were used in line with the data sharing agreement with NHS Digital. HC, SR and KR had full access to the data, which was extracted for analysis in February 2020.

In patients aged ≥ 18 years, we included all first unplanned admissions, with a primary diagnosis code of heart failure (ICD10 I50.0, I50.1, I50.9, I11.0, I13.2, I13.0) occurring between 1st January 2002 and 31^st^ December 2018. The first HF admission per patient was selected as the index admission, to avoid dominance by HF patients who have multiple admissions. Transfers between hospitals were identified from discharge information and linked, to identify a final discharge date associated with each index admission. We cleaned the data by excluding patients who had multiple admissions starting on the same day, patients for whom we could not infer a final discharge date, patients who had additional admissions after their date of death, and patients with unknown age or sex (Figure S1).

#### 2.2 Socioeconomic status

The patient level Index of Multiple Deprivation (IMD) 2010 was used as a measure of socioeconomic status. The IMD 2010 combines seven weighted scores relating to different domains of deprivation (income, employment, health and disability, education, skills and training, barriers to housing and services, living environment and crime), to generate an index score of deprivation, covering small housing areas in England [22]. The score was ranked into quintiles, ranging from least affluent (quintile 1) to most affluent (quintile 5).

#### 2.3 Ethnicity

Ethnicity was categorised into 5 distinct groups for the analyses, reflecting the most prevalent ethnic groups in the 2011 census in England and Wales [23], as follows: White, South Asian, Black, ‘Mixed/other’ and ‘Unknown’. South Asian included Pakistani, Indian, Bangladeshi and other Asian ethnic groups such as Asian British while Black includes African, Caribbean and other Black groups such as Black British.

#### 2.4 Outcomes

First readmission within 30-days for HF (ICD10 I50.0, I50.1, I50.9, I11.0, I13.2, I13.0), other CVD (ICD-10 chapter 9, excluding HF) and non-CVD (remaining ICD-10 chapters).

#### 2.5 Baseline characteristics

We collected information on cardiovascular comorbidities (ischaemic heart disease (IHD), myocardial infarction (MI), hypertension, atrial fibrillation (AF)) and non-cardiovascular comorbidities (diabetes, chronic obstructive pulmonary disease (COPD), asthma, depression, cancer, chronic kidney disease, cerebrovascular accident (CVA), dementia, anaemia, arthrosis, rheumatoid arthritis). We used HES ICD-10 codes in any position, to ascertain comorbidities listed in the index admission.

Information on cardiac interventions during the index admission was extracted including whether or not the patient was under cardiologist care during their hospital admission (cardiology speciality) and the presence or absence of coronary artery bypass grafting, percutaneous coronary intervention (PCI), implantable cardioverter defibrillator (ICD), cardiac resynchronisation therapy (CRT) and permanent pacemaker (see Table S1 for full ICD-10 code list).

#### 2.6 Statistical analysis

Baseline characteristics were first presented by calendar period of index HF admission using two summary 5-year time windows at the beginning (2002-2006) and end (2014-2018) of our study time period, as number (%) or median [25th, 75^th^ percentile]. Readmission proportions for any cause within 30-days, for survivors of the index admission, were calculated for each calendar year and for the two 5-year summary time windows. Readmission proportions were then stratified by HF, ‘other-CVD’ and ‘non-CVD’. As data were over dispersed, quasipoisson models were used to estimate readmission proportions. Age was entered as a cubic spline with 3 knots to account for the non-linear effect of age. We also categorised age into 10 year bands, with the band (70-79) used as a reference category. To identify whether trends differed by sex, ethnicity and socioeconomic status, we entered each group into the models individually and with an interaction term between the group and calendar year. Models were built in R and used to estimate predicted proportions of readmissions for each group at the mean age 79 years. Average proportions were estimated for each calendar year of admission and for the two summary time windows. The same models were then built in Stata SE version 15 and the Margins command was used to estimate the average change in proportions per year with 95% confidence intervals. The top ten ICD-10 codes for each admission type were identified. Next, to identify potential predictors of each readmission type, we used a backwards stepwise logistic model, entering the age spline and all patient characteristics, comorbidities and procedures. Variables were removed based on AIC. The effect of age was further investigated by plotting age against predictive probabilities.

We calculated in-hospital deaths for the two summary time-windows and excluded these individuals from the readmission analysis. To account for the competing risk of death, we estimated readmission proportions only in the survivors of the index admission and estimated proportions of 30-day deaths occurring and a composite of 30-day readmission or death.

### 3 Role of the funding source

This publication presents independent research funded by the National Institute for Health Research (NIHR). The study sponsors had no role in the design and conduct of the study; collection, management, analysis, and interpretation of the data; preparation, review, or approval of the manuscript; and decision to submit the manuscript for publication. The views expressed are those of the author(s) and not necessarily those of the NHS, the NIHR or the Department of Health and Social Care.

### 4 Results

#### 4.1 Study population

There were 698,983 index hospital admission for HF between 1^st^ January 2002 and 31^st^ December 2018: median age 81 years [IQR 73, 87]; 351,821 (50%) male; 572,907 (82%) White; 22,008 (3.1%) South Asian; 12,169 (1.7%) Black. Socioeconomic status ranged from most affluent (16%) to least affluent (22%). Most prevalent comorbidities were hypertension (46%), AF (43%), diabetes (27%), CKD (15%) and COPD (14%), all of which increased substantially over time (Table 1). Prevalence of procedures was low (<1%) with some increases over time for PCI, ICD, CRT and pacemaker implantation.

**Table 1:** Patient characteristics by time period

	ALL YEARS (N=698,983)	2002-2006 (N=217,258)	2014-2018 (N=220,934)
Age	81 [73, 87]	80 [72, 86]	81 [73, 87]
Male	351,821 (50.3)	107,437 (49.5)	113,778 (51.5)
Female	347,162 (49.7)	109,821 (50.5)	107,156 (48.5)
*Ethnicity groups*			
White	572,907 (82.0)	157,342 (72.4)	189,034 (85.6)
South Asian	22,008 (3.1)	5,001 (2.3)	8,487 (3.8)
Black	12,169 (1.7)	2,618 (1.2)	4,698 (2.1)
Other/mixed	12,540 (1.8)	2,524 (1.2)	5,188 (2.3)
Unknown	79,359 (11.4)	49,773 (22.9)	13,527 (6.1)
*Socioeconomic status*			
5 (least deprived)	114,799 (16.4)	32,791 (15.1)	38,274 (17.3)
4	134,320 (19.2)	40,086 (18.5)	43,691 (19.8)
3	143,582 (20.5)	44,611 (20.5)	45,155 (20.4)
2	146,992 (21.0)	47,224 (21.7)	45,102 (20.4)
1 (most deprived)	151,403 (21.7)	50,740 (23.4)	44,844 (20.3)
*Comorbidities*			
AF	297,039 (42.5)	65,458 (30.1)	114,931 (52.0)
Anaemia	29,076 (4.2)	5,321 (2.4)	13,193 (6.0)
Arthrosis	45,744 (6.5)	6,752 (3.1)	22,816 (10.3)
Asthma	43,335 (6.2)	9,714 (4.5)	16,642 (7.5)
Cancer	37,397 (5.4)	8,695 (4.0)	14,417 (6.5)
CKD	106,698 (15.3)	12,286 (5.7)	63,687 (28.8)
COPD	98,616 (14.1)	24,595 (11.3)	37,635 (17.0)
CVA	12,681 (1.8)	4,090 (1.9)	3,811 (1.7)
Dementia	32,510 (4.7)	5,589 (2.6)	14,736 (6.7)
Depression	17,488 (2.5)	2,193 (1.0)	9,413 (4.3)
Diabetes	188,913 (27.0)	46,516 (21.4)	70,726 (32.0)
Hypertension	321,457 (46.0)	64,051 (29.5)	126,115 (57.1)
Rheum arthritis	13,019 (1.9)	2,750 (1.3)	5,538 (2.5)
*Procedures*			
PCI	2794 (0.4)	530 (0.2)	1137 (0.5)
ICD	828 (0.1)	30* (0.01)	368 (0.2)
CABG	1766 (0.3)	517(0.2)	515 (0.2)
CRT	1969(0.3)	20*(0.01)	1077 (0.5)
Pacemaker	5285(0.8)	1183(0.5)	2267 (1.0)

Data are reported as number of patients (%) or median with 25th and 75th centiles. AF, atrial fibrillation; CKD, chronic kidney disease; COPD, chronic obstructive pulmonary disease; CVA, cerebrovascular accident; PCI, Percutaneous coronary intervention; ICD, internal cardiac defibrillator; CABG, Coronary artery bypass grafting; CRT, cardiac resynchronisation therapy.

All differences were statistically significant with p<0.001 except for CABG (p=0.763).

⁎ Indicates small numbers (< 100) that have been rounded up to the nearest 10

There were 120,888 (21%) 30-day readmissions for any cause: 6% HF; 3% other CVD; and 12% non-CVD causes. In hospital deaths occurred in 108,798 patients (16%) and a further 31,178 (5%) occurred by 30 days (Table 2).

**Table 2:** In hospital and 30-day outcomes

Outcome	Total	Early (2002-2006)	Late (2014-2018)
*Deaths*			
In hospital deaths	108798 (15.6)	42858 (19.7)	25581 (11.6)
30 day deaths	31178 (5.3)	8767 (5.0)	10872 (5.6)
*30-day readmissions*			
All readmissions	120888 (20.5)	32509 (18.6)	43348 (22.2)
HF readmissions	33441 (5.7)	9808 (5.6)	11726 (6.0)
Other CV readmissions	17517 (3.0)	5461 (3.3)	5298 (2.7)
Non CV readmissions	68669 (11.6)	16682 (9.6)	25988 (13.3)
*30-day readmission or death*	152,066 (25.8)	41,276 (23.7)	54,220 (27.8)

CV, cardiovascular. 30-day deaths and readmissions were calculated in survivors on the index admission.

#### 4.2 Readmission proportions and 16-year trends by age

**Fig. 1:**
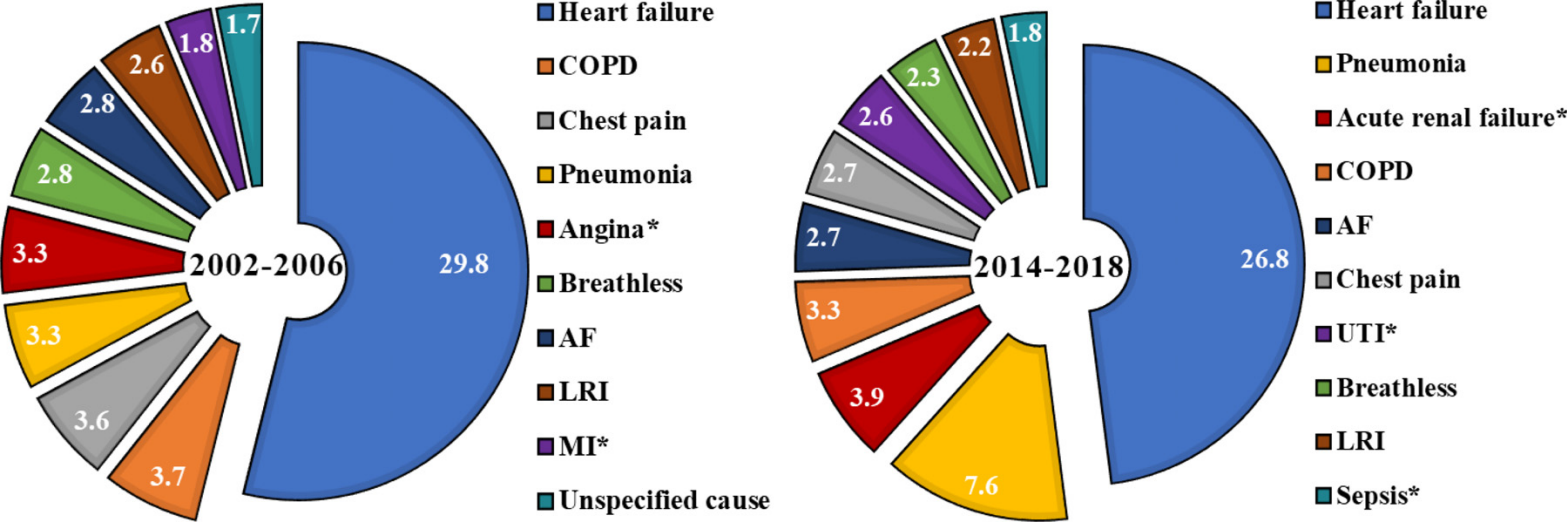
Top ten causes of readmission by time period. The charts show the percentages of specific causes of admission, for each time period. Percentages are calculated using the number of readmissions for each of the causes, out of the total number of readmissions. Only the top ten are displayed and they are presented in descending order of prevalence. Causes with * are unique to that time period.

**Fig. 2:**
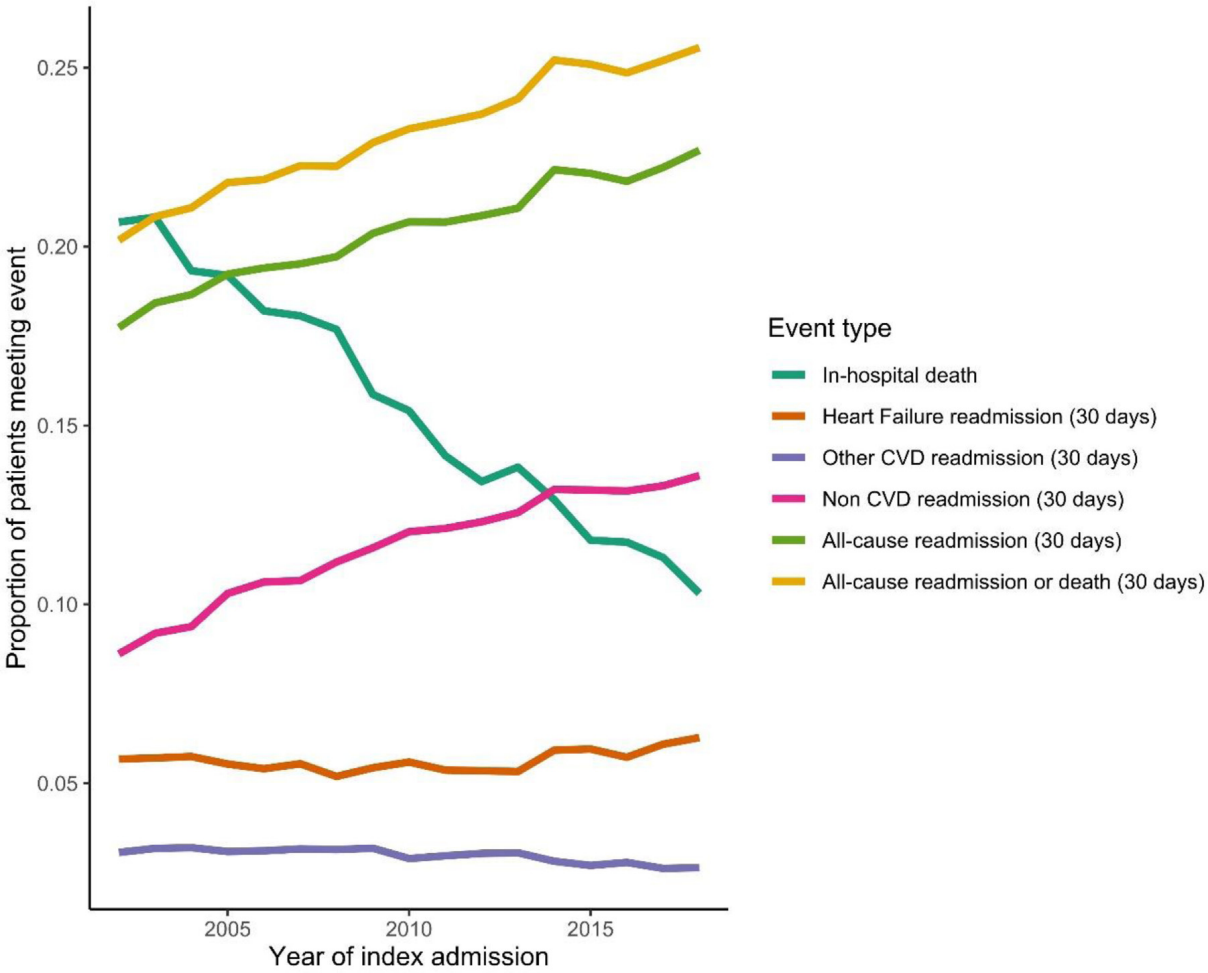
Plot of trends in probability of in-hospital death, 30-day readmission (split by cause) and/or 30-day death over time. The figure shows the predicted proportions of events for all index admissions with HF, during the study time window (1st January 2002 and 31st December 2018). All predictions are made at the mean age 79 years. With the exception of in-hospital deaths, all proportions are estimated using the sum of first event within 30-days of discharge from hospital as the numerator and all live discharges as the denominator.

Between 2002-2006 and 2014-2018, age adjusted all-cause readmissions increased from 19% to 22%, an average increase of 1.4% (95% CI 1.3, 1.5) per annum (Table 3). The highest proportions were in the youngest (≤ 39 years, 21%) and oldest groups (≥ 90 years; 22%). By cause, HF and ‘other CVD’ readmission proportions were similar across different age groups (approx. 6% and 3% respectively) and remained relatively stable over time. Highest overall readmission proportions and increases over time were for non-CVD causes, increasing from 10% (10, 10) to 13% (13, 14) an average increase of 2.6% (2.4, 2.7) per annum. Risk of readmission for Non-CVD causes by age followed the same pattern as all-cause admissions, with highest overall proportions and steepest increase in proportions in the youngest and oldest age groups (Table 3). Out of the readmitted patients in the early time period, non-CVD causes constituted 50% of the top ten readmission causes (COPD, pneumonia, breathlessness, lower respiratory infection and unspecified causes). In the later time period non-CVD causes constituted 70% of the top ten causes (pneumonia, acute renal failure, COPD, UTI, respiratory infection and sepsis) (Figure 1). Over the same time-period, in hospital deaths reduced from 20% to 12%, at a rate of 0.7% per annum, whilst 30-days deaths remained stable (Table 2 and Figure 2).

**Table 3:** Predicted proportions of 30 day re-admissions by age and calendar year

	All years	2002 - 2006	2014 - 2018	Relative diff. (%)^a^ P Interaction^b^		Average annual percent change per year (95% CI)^c^
All admissions						
All	0.21 (0.20, 0.21)	0.19 (0.19, 0.19)	0.22 (0.22, 0.23)	18.1	Ref	1.4 (1.3,1.5)
<= 39	0.21 (0.17, 0.26)	0.21 (0.19-0.23)	0.21 (0.19-0.22)	-3.8	**0.003**	-0.4 (-1.7,1.0)
40-49	0.18 (0.159, 0.214)	0.17 (0.16-0.18)	0.19 (0.18-0.20)	11.6	0.105	0.8 (-0.1,1.7)
50-59	0.18 (0.168, 0.200)	0.17 (0.16-0.17)	0.20 (0.19-0.21)	18.5	0.413	1.3 (0.8,1.8)
60-69	0.19 (0.182, 0.202)	0.18 (0.18-0.18)	0.20 (0.20-0.21)	14.5	0.051	1.1 (0.8,1.4)
70-79	0.20 (0.195, 0.209)	0.19 (0.18-0.19)	0.22 (0.22-0.22)	18.9	Ref	1.5 (1.3,1.7)
80-89	0.21 (0.206, 0.218)	0.19 (0.19-0.20)	0.23 (0.23-0.23)	18.0	0.406	1.4 (1.2,1.5)
>= 90	0.22 (0.204, 0.226)	0.19 (0.19-0.19)	0.23 (0.23-0.24)	23.8	0.118	1.7 (1.4,2.0)
	**HF admissions**					
All	0.06 (0.06, 0.06)	0.06 (0.06, 0.06)	0.06 (0.06, 0.06)	5.4	Ref	0.5 (0.3,0.7)
<= 39	0.06 (0.06, 0.06)	0.05 (0.04, 0.07)	0.05 (0.04, 0.07)	3.8	0.713	-0.1 (-2.8,2.5)
40-49	0.06 (0.04, 0.07)	0.05 (0.04, 0.06)	0.05 (0.05, 0.06)	17.8	0.313	1.5 (-0.2,3.3)
50-59	0.05 (0.04, 0.06)	0.05 (0.04, 0.06)	0.06 (0.05, 0.06)	16.3	0.364	1.0 (0.0,2.0)
60-69	0.05 (0.05, 0.06)	0.06 (0.05, 0.06)	0.06 (0.05, 0.06)	3.6	0.994	0.4 (-0.2,1.1)
70-79	0.06 (0.05, 0.06)	0.06 (0.05, 0.06)	0.06 (0.06, 0.06)	5.5	Ref	0.5 (0.0,1.0)
80-89	0.06 (0.06, 0.06)	0.06 (0.05, 0.06)	0.06 (0.06, 0.06)	3.6	0.766	0.3 (-0.1,0.8)
>= 90	0.06 (0.05, 0.06)	0.05 (0.05, 0.06)	0.06 (0.06, 0.06)	11.5	0.246	0.9 (0.3,1.6)
	**Other CV**					
All	0.03 (0.03, 0.03)	0.03 (0.03, 0.03)	0.03 (0.03, 0.03)	-12.5	Ref	-1.1 (-1.4,-0.8)
<= 39	0.04 (0.03, 0.05)	0.05 (0.04, 0.07)	0.03 (0.03, 0.04)	-35.8	0.136	-3.3 (-6.2,-0.3)
40-49	0.04 (0.03, 0.04)	0.04 (0.03, 0.05)	0.04 (0.03, 0.04)	-10.0	0.993	-0.8 (-2.8,1.1)
50-59	0.03 (0.03, 0.03)	0.03 (0.03, 0.04)	0.04 (0.03, 0.04)	9.4	0.104	0.3 (-0.8,1.5)
60-69	0.03 (0.03, 0.03)	0.03 (0.03, 0.04)	0.03 (0.03, 0.03)	-11.8	0.575	-1.1 (-1.9,-0.3)
70-79	0.03 (0.03, 0.03)	0.03 (0.03, 0.03)	0.03 (0.03, 0.03)	-12.5	Ref	-0.8 (-1.4,-0.3)
80-89	0.03 (0.03, 0.03)	0.03 (0.03, 0.03)	0.03 (0.02, 0.03)	-19.4	**0.038**	-1.6 (-2.1,-1.1)
>= 90	0.02 (0.02, 0.03)	0.03 (0.02, 0.03)	0.02 (0.02, 0.03)	-4.0	0.361	-0.3 (-1.2,0.6)
	**Non CV**					
All	0.12 (0.11, 0.12)	0.10 (0.10, 0.10)	0.13 (0.13, 0.14)	36.7	Ref	2.6 (2.4,2.7)
<= 39	0.11 (0.07, 0.17)	0.10 (0.09, 0.12)	0.11 (0.10, 0.13)	11.8	0.084	0.8 (-1.1,2.7)
40-49	0.09 (0.07, 0.13)	0.09 (0.08, 0.10)	0.10 (0.09, 0.11)	18.8	**0.029**	1.1 (-0.2,2.4)
50-59	0.09 (0.08, 0.11)	0.08 (0.08, 0.09)	0.10 (0.10, 0.11)	26.8	0.065	1.9 (1.1,2.7)
60-69	0.10 (0.09, 0.11)	0.09 (0.08, 0.09)	0.12 (0.11, 0.12)	34.9	0.33	2.4 (1.9,2.9)
70-79	0.11 (0.11, 0.12)	0.09 (0.09, 0.10)	0.13 (0.13, 0.14)	40.4	Ref	2.8 (2.4,3.1)
80-89	0.12 (0.12, 0.13)	0.10 (0.10, 0.11)	0.14 (0.14, 0.15)	37.9	0.705	2.7 (2.4,3.0)
>= 90	0.13 (0.12, 0.14)	0.11 (0.10, 0.11)	0.15 (0.14, 0.15)	37.0	0.539	2.6 (2.1,3.1)

All proportions are estimated using the sum of first readmissions within 30-days of discharge from hospital as the numerator and all live discharges as the denominator. Live discharges are from all survivors of the index admission with HF, during the study time window (1st January 2002 and 31st December 2018).

CV, cardiovascular; Relative diff., relative difference; CI, confidence interval.

a relative percentage difference in admission proportions between the first and second diagnosis time periods, calculated by 100*([time-period 2 – time period 1] / time-period 1].

b P value for the difference in trend lines between groups, compared to the reference group. Estimated by fitting an interaction term between calendar year and group in the quasipoisson models also containing age.

c Average annual percentage change in rates (per 100 person-years) for each increasing year of index HF admission

#### 4.3 Readmission proportions and 16-year trends by sex, socioeconomic status and ethnicity

Men had a similar proportion of all-cause readmissions (21%; 20,21) to women (20%, 20,20) with similar increases over time (interaction p=0.3) and patterns were similar for all readmission causes (S2 Table). The least affluent group had a higher proportion of all-cause readmissions (22%, 22,22) than the most affluent (19%; 19,20), a gap which remained constant over time. This gap was explained by non-CVD causes which increased from 9 to 12 % for the most affluent and 11 to 15% for the least affluent. Proportions of all-cause readmissions were similar by ethnicity in the earlier time-period, but they were higher for South Asian and Black groups (both 24%; 23, 25), compared to the White group (22%, 22,23) in the later time period. Whilst the proportion of readmissions for HF remained stable for all other groups, the Black group experienced an increase in HF readmissions, from 6% to 9% over time (interaction p=0.03). The South Asian group also experienced a slightly higher annual increase in non-CVD readmissions (3.3% per annum) that than the White (2.3% per annum) and Black groups (2.2% per annum) (interaction p=0.04).

Trends remained similar for the composite of 30-day readmission or death (Table S3)

#### 4.4 Predictors of readmission

**Fig. 3:**
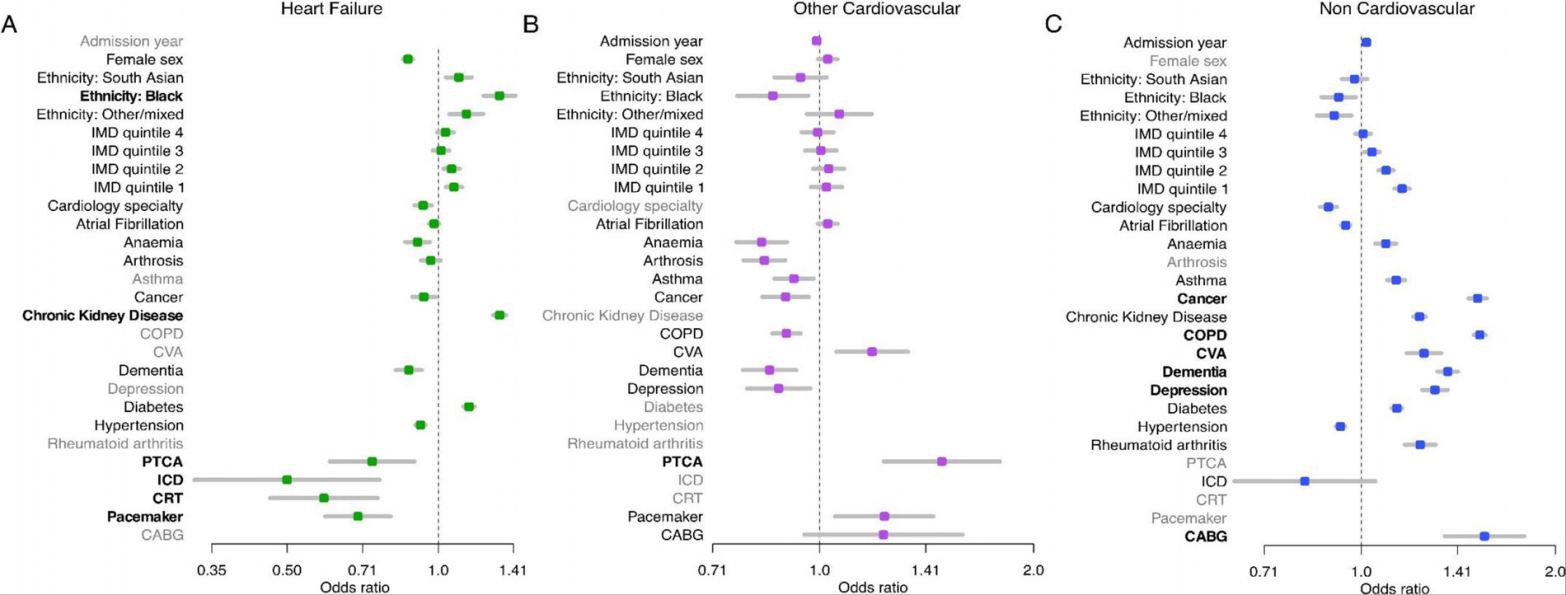
Predictors of readmission by cause. The graphs show the result of logistic models for each readmission cause with all of the variables. Variables that did not contribute sufficiently to the fit (based on AIC) with a backwards step procedure were removed (shaded in grey) and the remaining odds ratios were plotted. Variables are in bold if the point estimate for the odds ratio is <0.8 or > 1.25. Age spline coefficients were removed to ease interpretation.

In the multivariable models, the strongest predictors for readmissions were as follows; readmissions for HF: Black ethnicity (OR, 1.32; 95% CI 1.23, 1.42), CKD (OR 1.32; 1.29, 1.36), PCI (OR 0.74; 0.61,0.90), ICD (OR 0.50; 0.33,0.76), CRT (OR 0.59; 0.46,0.76), pacemaker (OR 0.69; 0.60,0.80) and cardiology speciality (OR 0.93; 0.90,0.97)); readmissions for ‘other CVD’: CVA (OR 1.19; 1.06,1.33), CABG (OR 1.23; 0.95,1.59), Pacemaker (OR 1.23; 1.05,1.44) and PCI (OR 1.49; 1.23,1.79); readmissions for non-CVD: Cancer (OR 1.51; 1.47,1.56), COPD (OR 1.53; 1.49,1.56)), CVA (OR 1.25; 1.18,1.33), dementia (OR 1.36; 1.31,1.41), depression (OR 1.30; 1.24,1.36), CABG (OR 1.55; 1.35,1.79) and cardiology speciality (OR 0.89; 0.86,0.92) (Figure 3, Table S4). In terms of age, overall proportions of readmission for any cause and for non-CVD causes, reduced to and increased from, around 60 years of age Figure S2).

### 5 Discussion

Despite readmission reduction policies, the proportion of people with HF readmitted within 30-days has increased; a change that was driven by non-CVD conditions. Readmissions for HF and other CVD causes have remained stable, but there were differences among population groups. Readmissions for HF increased in Black groups; a diverging trend that culminated in a significantly higher risk in Black groups, compared to White groups, in recent years. We also noted a trend for more rapid increases in readmission proportions for non-CVD causes in the South Asian group compared to White or Black groups, and the least affluent group had a significantly higher risk of readmission for non-CVD causes compared to the most affluent group.

To our knowledge this is the first study of its scale to report contemporary national data on 30-day readmissions in England by age, sex, ethnicity and socioeconomic status. By using a multi-ethnic database linked to socioeconomic and death data over 16-years, we were able to identify potentially important patient characteristics and trends, but it is difficult to disentangle these findings from those relating to care quality and provision across inpatient, transitional and post discharge pathways. Whilst group differences remained after accounting for a range of patient and service factors, we were not able to adjust for case mix. The number of South Asian and Black patients were lower than expected from national UK census data [23], representing only a small portion of the total patients within our study, and so may not be representative of the population. Also we did not have prescription or echocardiograph data so we could not investigate changes to prescriptions over time or any differences by HF phenotypes. The quality of HES data has been continually improving, with the introduction of payment by results in 2003/04 a major driving factor. Incremental changes to classification systems (ICD10 and OPCS4) could also have a small effect on trends observed in HES data. It is possible that improved data quality could have resulted in more accurate longitudinal linking of spells to the same patient and hence accentuate any increase in readmission proportions. However, we would expect any such effects to apply consistently across patient groups and be independent of readmission cause. We have provided national 30-day readmission demographics indicating key targets, but these require further consideration at a local level.

Reducing the high cost and burden of early readmissions after HF admission has become a health policy priority of recent years, yet prior trend data are conflicting and demographic data are scarce. Epidemiological reports have been conducted mostly at an aggregate level at a single time point, which doesn't take account of differences in cause-specific readmissions over time. Thirty-day readmissions for any cause increased from 19 to 22% over the time period of our study. These proportions are slightly higher than the 18% reported in a previous UK study [15], lower than in US studies [24,25], and similar to proportions reported in Spain [13]. The prior UK study used a single ICD code to define incident HF admissions and the US studies included people > 65 years of age, which may explain the discrepancies. In terms of trends, a UK HF study reported 30-day readmissions for any cause to be stable, at approximately 18%, for the decade 2006 to 2016 [26]. However, this study combined elective and unplanned HF admissions and repeat admissions for individual patients. Readmissions following an elective admission were fewer and reduced over time, so likely masked any increase in readmissions following unplanned admission. Inclusion of day-case patients and patients with multiple admissions, whom are often younger [15], may also explain the lower risk in the prior study. Our report of increasing readmissions is in contrast to reports of slight reductions in readmissions for any cause and for HF and stable readmissions for non-CVD causes, reported in the US and Canada [[10], [11], [12],^27^]. Our findings more closely mirror reports of increasing readmissions for non-CVD causes in Europe [13,14], and adds new key evidence on differences among different patient groups with HF.

Large studies of Medicare beneficiaries reported that the spectrum of readmission diagnoses did not vary by patient characteristics [12,25]. These studies were conducted over a small time window and whilst one study reported absolute proportions of readmissions for HF to be higher in Black groups than in White groups, adjusted analyses in both studies focused only on readmissions for any cause, where no significant associations were found. By using longitudinal data and cause-specific readmissions, we found that initial similarity between ethnic groups diverged over time with a higher risk of readmissions for HF in Black groups in recent years. This difference remained after adjustment for age, comorbidities, health care interventions and socio-economic status. Higher risk for Black groups was also identified for readmissions for any cause in Emory Healthcare data, taking account of local processes [28].

In-hospital deaths were high (16% overall), which likely reflects the high mortality risk following a new diagnosis of HF. Prior evidence in England has shown that a high proportion of patients are diagnosed with HF during an index HF admission [6]. Although 30-day mortality following discharge from hospital remained stable in our study, part explanation for increased readmissions in England may be explained by the improvement in hospital survival after HF admission (8% reduction in in-hospital mortality over time, compared to only 1% in the US and Canada). However, given that the increase in readmissions was driven by non-CVD causes, a contributing factor is likely to be the increasing prevalence of non-CVD comorbidities over the same time period, particularly in the least affluent and South Asian groups [29]. The increasing HF readmissions in the Black group may reflect the increasing prevalence of hypertensive related HF with preserved ejection fraction in this group [30].

Reasons for early readmission are diverse and contributions are from patient complexity, self-care efficacy and health service factors. Public reporting of hospital readmission proportions was introduced in England in 2001, followed by financial incentives in 2011 [9]. The policy focus in England is on avoidable unplanned readmissions for any cause within 30 days [31], in contrast with policies in targeting specific causes in Germany [32] and the US. [33] Non-payment for readmissions are based on hospital-specific readmission rates, a process that acknowledges different case mixes across Hospital Trusts, with the underpayment being diverted into post discharge care improvements. Despite these long standing incentives, we found an increase in readmissions for HF patients in England. This increase may in part be explained by the adjacent success of other initiatives such as specialist HF services providing care, on an outpatient basis, to patients with chronic HF. In this context the admitted patients are likely to be sicker with a higher risk of readmission. Against this backdrop of a potentially more severe admitted group of HF patients, improvements in prescribing and fast up titration of HF drug therapies, potentially explains the reduction in in-hospital death and the stability of readmissions for HF. Interestingly, whilst cardiac procedures during admission significantly reduced the risk of readmission for HF, their implementation only increased slightly, which may be reflective of improvements in pharmacological prescribing or increasing complexity of HF patients over time. However, the increase in readmissions for non-CVD causes is concerning and this risk was higher in non-cardiology settings, pointing to the need for better comorbidity management and specialist review. Reducing the high and increasing rate of non-cardiovascular readmissions will require a multidisciplinary approach to specialist cardiology and non-cardiovascular discharge planning and for readmission prevention strategies to realign with the increasing complexity of HF patients.

Despite policy drive to reduce costly and burdensome readmissions in HF, 30-day readmissions are increasing. This change is mostly driven by non-cardiovascular causes and impacts the least affluent and ethnic minority groups the most. The drive to reduce the burden and costs of early readmissions in HF and to develop more effective systems of care will require close consideration of patient factors and a multidisciplinary approach to specialist non-cardiovascular care.

### 6 Funding

CL is funded by National Institute for Health Research (NIHR) Advanced fellowship [reference: NIHR 300111] for this research project. KK and FZ are funded with an unrestricted educational grant from the NIHR ARC East Midlands and the NIHR Leicester BRC and this study is a collaboration between NIHR ARC East Midlands and NIHR ARC West Midlands

### 7 Data sharing agreement

HC had full access to all the data in the study and takes responsibility for the integrity of the data and the accuracy of the data analysis. Linked HES and ONS data may be obtained from NHS Digital via the Data Access Request Service (DARS) and are not publically available.

### 8 Author contributions

CL: conception, design, interpretation of data, writing draft; HC/SR/KR: analysis, data verification, critical review; IS/MD/LB: conception, critical review; FZ: conception, design, critical review; RL: acquisition of data, critical review; KK: conception, acquisition of data, critical review

## Declaration of Competing Interest

Dr. Khunti reports personal fees from Amgen, Abbott, AstraZeneca, Bayer, NAPP, Lilly, Merck Sharp & Dohme, Novartis, Novo Nordisk, Roche, Berlin-Chemie AG / Menarini Group, Boehringer-Ingelheim, Sanofi-Aventis and Servier, served as a board member at Astrazeneca, Lilly, Merck Sharp & Dohme, Novo Nordisk, Sanofi-Aventis, reports grants from AstraZeneca, Novartis, Novo Nordisk, Sanofi-Aventis, Lilly, Servier, Pfizer, Boehringer Ingelheim and Merck Sharp & Dohme, outside the submitted work; Dr. Zaccardi reports and Speaker fees from Napp Pharmaceutical and Boehringer Ingelheim; Dr. Davies reports personal fees from Novo Nordisk, personal fees from Sanofi, personal fees from Eli Lilly, personal fees from Boehringer Ingelheim, personal fees from AstraZeneca, personal fees from Gilead Sciences Ltd, personal fees from Janssen, personal fees from Lexicon, personal fees from Napp Pharmaceuticals, personal fees from Takeda Pharmaceuticals International Inc., grants from AstraZeneca, grants from Novo Nordisk, grants from Boehringer Ingelheim, grants from Janssen, grants from Sanofi, outside the submitted work. All other authors declare no competing interests.

## Appendix Supplementary materials

Image, application 1
