# Supplementary material for: Trends in 30-day readmissions following hospitalisation for heart failure by sex, socioeconomic status and ethnicity
Source: eClinicalMedicine. 2021 Jul 14;38:101008. doi: 10.1016/j.eclinm.2021.101008 (PMC8283308; doi:10.1016/j.eclinm.2021.101008)
Supplement: Supplementary file 1 [file mmc1.docx]

**16-year trends in 30-day cause specific readmissions following hospitalisation for heart failure in England by sex, socioeconomic status and ethnicity.**

**Supplementary File**

Lawson C, Crothers HE, Remsing S, Zaccardi F, Bernhardt L, Reeves K, Lilford RJ, Khunti K.

- Claire A Lawson; NIHR Advanced Fellow, Cardiovascular Sciences Department and Leicester Real World Evidence Unit, University of Leicester.
- Hannah Crothers; University of Birmingham
- Sandra Remsing; University of Birmingham
- Francesco Zaccardi; Clinical Research Fellow, Leicester Real World Evidence Unit, University of Leicester.
- Lizelle Bernhardt; NIHR Doctoral Fellow, University of Leicester
- Katharine Reeves; University of Birmingham
- Richard Lilford, University of Birmingham
- Kamlesh Khunti; Professor of Primary Care Diabetes & Vascular Medicine, Diabetes Research Centre, University of Leicester, UK

**Corresponding author:**

Dr C. A. Lawson

University of Leicester, Leicester, Leicestershire

LE5 4PW, England, UK

Email: [cl417@leicester.ac.uk](mailto:cl417@leicester.ac.uk)

Telephone: +44 (0) 4782 709 590

Table of Contents

[S1 Table ICD-10 Codes for comorbidities and procedures 3](#_Toc61622853)

[S2 Table: Predicted rates of 30 day re-admissions by population groups and calendar year 4](#_Toc61622854)

[S3 Table: Predicted rates of 30 day re-admissions or death by population groups and calendar year 6](#_Toc58056515)

[S4 Table: Associations between patient and clinical characteristics and readmissions by cause 9](#_Toc58056516)

S1 Figure: Cohort selection flowchart 11

S2 Figure: Effect of age on probability of readmission 12

| S1 Table ICD-10 Codes for comorbidities and procedures | |
| --- | --- |
| Condition/Procedure | Codes ICD10 / *OPCS 4* |
| Hypertension | I10 – I 15 |
| Atrial Fibrillation | I48 |
| Diabetes | E10 – E14 |
| COPD | J44 |
| Asthma | J45 |
| Depression | F32 – F33 |
| Cancer | C00 – C97 |
| Chronic Kidney Disease | N18 |
| Cerebrovascular Accident | I60 – I64 & I69 |
| Dementia | F00 – F03 |
| Anaemia | D50 |
| Arthrosis | M15 – M19 |
| Rheumatoid arthritis | M05 – M06 |
| Coronary artery bypass grafting | *K40 - K46* |
| Percutaneous coronary intervention | *K49, K50, K75* |
| Cardioverter defibrillator insertion | *K59.1, K59.2, K72.1* |
| Cardiac resynchronisation therapy | *K60.7, K61.7, K59.6* |
| Pacemaker insertion | *K60.1, K60.5, K60.6, K61.1, K61.5, K61.6* |

| S2 Table: Predicted rates of 30 day re-admissions by population groups and calendar year | | | | | | |
| --- | --- | --- | --- | --- | --- | --- |
|  | Proportion of patients with 30 day readmission | | | Relative diff. (%)^a^ | P interaction^b^ | Average annual percent change per year (95% CI)^c^ |
|  | **All years** | **2002 - 2006** | **2014 - 2018** |  | |  |
|  | **All** | | | | | |
| All | 0.21 (0.20, 0.21) | 0.19 (0.19, 0.19) | 0.22 (0.22, 0.23) | 18.1 |  | 1.4 (1.3,1.5) |
| Men | 0.21 (0.21, 0.21) | 0.19 (0.19, 0.20) | 0.23 (0.22, 0.23) | 17.1 | Ref | 1.3 (1.2,1.5) |
| Women | 0.20 (0.20, 0.20) | 0.18 (0.18, 0.19) | 0.22 (0.22, 0.22) | 19.1 | 0.314 | 1.4 (1.3,1.6) |
| Most affluent (Q5) | 0.19 (0.19, 0.20) | 0.18 (0.17, 0.18) | 0.21 (0.21, 0.21) | 18.6 | Ref | 1.4 (1.1,1.7) |
| Least affluent (Q1) | 0.22 (0.22, 0.22) | 0.20 (0.20, 0.20) | 0.24 (0.24, 0.24) | 20.0 | 0.951 | 1.4 (1.2,1.7) |
| White | 0.21 (0.21, 0.21) | 0.19 (0.19, 0.20) | 0.22 (0.22, 0.23) | 14.9 | Ref | 1.2 (1.1,1.3) |
| South Asian | 0.22 (0.21, 0.23) | 0.19 (0.18, 0.20) | 0.24 (0.23, 0.25) | 21.6 | 0.163 | 1.7 (1.1,2.3) |
| Black | 0.22 (0.21, 0.23) | 0.20 (0.19, 0.22) | 0.24 (0.23, 0.25) | 17.9 | 0.641 | 1.4 (0.6,2.2) |
| Mixed/other | 0.21 (0.20, 0.22) | 0.21 (0.20, 0.23) | 0.21 (0.20, 0.23) | 1.4 | **0.021** | 0.3 (-0.6,1.1) |
| Unknown | 0.16 (0.16, 0.17) | 0.16 (0.16, 0.16) | 0.18 (0.17, 0.18) | 10.1 | **0.024** | 0.7 (0.4,1.1) |
|  | **HF** | | | | | |
| All | 0.06 (0.06, 0.06) | 0.06 (0.06, 0.06) | 0.06 (0.06, 0.06) | 5.4 |  | 0.5 (0.3,0.7) |
| Men | 0.06 (0.06, 0.06) | 0.06 (0.06, 0.06) | 0.06 (0.06, 0.06) | 5.1 | Ref | 0.4 (0.1,0.6) |
| Women | 0.05 (0.05, 0.05) | 0.05 (0.05, 0.05) | 0.06 (0.05, 0.06) | 7.8 | 0.279 | 0.6 (0.3,0.9) |
| Most affluent (Q5) | 0.05 (0.05, 0.06) | 0.05 (0.05, 0.06) | 0.06 (0.05, 0.06) | 1.9 | Ref | 0.3 (-0.3,0.8) |
| Least affluent (Q1) | 0.06 (0.06, 0.06) | 0.06 (0.06, 0.06) | 0.06 (0.06, 0.07) | 10.5 | 0.46 | 0.5 (0.1,1.0) |
| White | 0.06 (0.06, 0.06) | 0.06 (0.06, 0.06) | 0.06 (0.06, 0.06) | 3.6 | Ref | 0.2 (0.0,0.4) |
| South Asian | 0.07 (0.06, 0.07) | 0.06 (0.06, 0.07) | 0.07 (0.06, 0.07) | 4.7 | 0.955 | 0.3 (-0.9,1.4) |
| Black | 0.08 (0.07, 0.08) | 0.06 (0.06, 0.07) | 0.09 (0.08, 0.09) | 32.8 | **0.027** | 1.8 (0.4,3.2) |
| Mixed/other | 0.07 (0.06, 0.07) | 0.06 (0.06, 0.07) | 0.07 (0.06, 0.07) | 3.1 | 0.716 | 0.5 (-1.0,2.0) |
| Unknown | 0.05 (0.05, 0.05) | 0.05 (0.05, 0.05) | 0.05 (0.05, 0.05) | 4.2 | 0.969 | 0.2 (-0.5,0.9) |
|  | **Other CV** | | | | | |
| All | 0.03 (0.03, 0.03) | 0.03 (0.03, 0.03) | 0.03 (0.03, 0.03) | -12.5 |  | -1.1 (-1.4,-0.8) |
| Men | 0.03 (0.03, 0.03) | 0.03 (0.03, 0.03) | 0.03 (0.03, 0.03) | -9.7 | Ref | -0.8 (-1.2,-0.4) |
| Women | 0.03 (0.03, 0.03) | 0.03 (0.03, 0.03) | 0.03 (0.03, 0.03) | -15.6 | **0.046** | -1.4 (-1.8,-1.0) |
| Most affluent (Q5) | 0.03 (0.03, 0.03) | 0.03 (0.03, 0.03) | 0.03 (0.03, 0.03) | -9.7 | Ref | -0.8 (-1.6,-0.1) |
| Least affluent (Q1) | 0.03 (0.03, 0.03) | 0.03 (0.03, 0.03) | 0.03 (0.03, 0.03) | -15.6 | 0.388 | -1.2 (-1.8,-0.6) |
| White | 0.03 (0.03, 0.03) | 0.03 (0.03, 0.03) | 0.03 (0.03, 0.03) | -15.2 | Ref | -1.3 (-1.6,-0.9) |
| South Asian | 0.03 (0.03, 0.03) | 0.03 (0.03, 0.03) | 0.03 (0.02, 0.03) | -10.0 | 0.838 | -1.1 (-2.7,0.5) |
| Black | 0.03 (0.02, 0.03) | 0.03 (0.03, 0.04) | 0.02 (0.02, 0.03) | -29.0 | 0.256 | -2.5 (-4.8,-0.3) |
| Mixed/other | 0.03 (0.03, 0.04) | 0.04 (0.03, 0.05) | 0.03 (0.02, 0.03) | -30.8 | 0.195 | -2.6 (-4.7,-0.5) |
| Unknown | 0.03 (0.03, 0.03) | 0.03 (0.03, 0.03) | 0.03 (0.02, 0.03) | -7.4 | 0.303 | -0.8 (-1.7,0.2) |
|  | **Non CVD** | | | | | |
| All | 0.12 (0.11, 0.12) | 0.10 (0.10, 0.10) | 0.13 (0.13, 0.14) | 36.7 |  | 2.6 (2.4,2.7) |
| Men | 0.12 (0.12, 0.12) | 0.10 (0.10, 0.10) | 0.13 (0.13, 0.14) | 35.4 | Ref | 2.5 (2.3,2.7) |
| Women | 0.11 (0.11, 0.12) | 0.10 (0.10, 0.10) | 0.13 (0.13, 0.14) | 37.1 | 0.325 | 2.6 (2.4,2.8) |
| Most affluent (Q5) | 0.11 (0.11, 0.11) | 0.09 (0.09, 0.09) | 0.12 (0.12, 0.12) | 37.8 | Ref | 2.7 (2.3,3.1) |
| Least affluent (Q1) | 0.13 (0.12, 0.13) | 0.11 (0.11, 0.11) | 0.15 (0.15, 0.15) | 38.3 | 0.843 | 2.6 (2.3,2.9) |
| White | 0.12 (0.12, 0.12) | 0.10 (0.10, 0.10) | 0.14 (0.13, 0.14) | 32.4 | Ref | 2.3 (2.2,2.5) |
| South Asian | 0.12 (0.12, 0.13) | 0.10 (0.09, 0.10) | 0.14 (0.13, 0.15) | 48.4 | **0.036** | 3.3 (2.4,4.1) |
| Black | 0.12 (0.11, 0.12) | 0.10 (0.09, 0.11) | 0.13 (0.12, 0.14) | 26.7 | 0.765 | 2.2 (1.0,3.3) |
| Mixed/other | 0.11 (0.10, 0.12) | 0.10 (0.09, 0.11) | 0.12 (0.11, 0.13) | 19.0 | 0.163 | 1.5 (0.4,2.7) |
| Unknown | 0.08 (0.08, 0.09) | 0.08 (0.08, 0.08) | 0.10 (0.09, 0.10) | 22.5 | **0.01** | 1.7 (1.2,2.2) |
| All predictions are at the mean population age (79 years). All proportions are estimated using the sum of first readmissions within 30-days of discharge from hospital as the numerator and all live discharges as the denominator. Live discharges are from all survivors of the index admission with HF, during the study time window (1st January 2002 and 31st December 2018).  HF, heart failure; CV, cardiovascular; Relative diff., relative difference; CI, confidence interval.  a relative percentage difference in admission proportions between the first and second diagnosis time periods, calculated by 100*([time-period 2 – time period 1] / time-period 1].  b P value for the difference in trend lines between groups, compared to the reference group. Estimated by fitting an interaction term between calendar year and group in the quasipoisson models also containing age.  c Average annual percentage change in rates (per 100 person-years) for each increasing year of index HF admission | | | | | | |

| S3 Table: Predicted rates of 30 day re-admissions or death by population groups and calendar year | | | | | |
| --- | --- | --- | --- | --- | --- |
|  | **All admissions** | | | |  |
|  | *Proportion of patients with 30 day readmission or death* | | *Relative diff. (%* | *P interaction* | *Average annual percent change in readmission/death probability per year (95% CI)* |
|  | **2002 - 2006** | **2014 - 2018** |  | |  |
| All | | | | | |
| All | 0.210 (0.208, 0.212) | 0.247 (0.245, 0.249) | 17.6 |  | 1.4 (1.3,1.5) |
| <= 39 | 0.215 (0.196, 0.237) | 0.217 (0.200, 0.235) | 0.9 | **0.024** | 0.0 (-1.4,1.3) |
| 40-49 | 0.181 (0.169, 0.193) | 0.203 (0.193, 0.214) | 12.2 | 0.183 | 0.8 (-0.1,1.7) |
| 50-59 | 0.178 (0.172, 0.185) | 0.208 (0.201, 0.215) | 16.9 | 0.396 | 1.2 (0.7,1.7) |
| 60-69 | 0.194 (0.190, 0.198) | 0.219 (0.215, 0.224) | 12.9 | **0.025** | 1.0 (0.7,1.3) |
| 70-79 | 0.205 (0.202, 0.208) | 0.241 (0.238, 0.245) | 17.6 | Ref | 1.4 (1.2,1.6) |
| 80-89 | 0.222 (0.220, 0.225) | 0.263 (0.260, 0.265) | 18.5 | 0.858 | 1.4 (1.2,1.5) |
| >= 90 | 0.236 (0.231, 0.241) | 0.292 (0.287, 0.297) | 23.7 | **0.027** | 1.7 (1.4,2.0) |
| Men | 0.216 (0.213, 0.219) | 0.252 (0.250, 0.255) | 16.7 | Ref | 1.3 (1.2,1.4) |
| Women | 0.203 (0.200, 0.205) | 0.240 (0.237, 0.243) | 18.2 | 0.335 | 1.4 (1.2,1.5) |
| Most affluent (Q5) | 0.201 (0.197, 0.205) | 0.237 (0.233, 0.241) | 17.9 | Ref | 1.4 (1.2,1.6) |
| Least affluent (Q1) | 0.221 (0.217, 0.224) | 0.262 (0.257, 0.266) | 18.6 | 0.962 | 1.4 (1.1,1.7) |
| White | 0.216 (0.213, 0.218) | 0.249 (0.246, 0.251) | 15.3 | Ref | 1.2 (1.1,1.3) |
| South Asian | 0.206 (0.196, 0.217) | 0.250 (0.241, 0.259) | 21.4 | 0.232 | 1.5 (0.9,2.1) |
| Black | 0.216 (0.203, 0.231) | 0.252 (0.240, 0.264) | 16.7 | 0.873 | 1.2 (0.4,2.0) |
| Mixed/other | 0.224 (0.209, 0.239) | 0.230 (0.219, 0.241) | 2.7 | **0.024** | 0.3 (-0.5,1.2) |
| Unknown | 0.183 (0.179, 0.187) | 0.205 (0.198, 0.211) | 12.0 | 0.067 | 0.9 (0.6,1.2) |
| HF | | | | | |
| All | 0.0930 (0.0915, 0.0944) | 0.0998 (0.0982, 0.1013) | 6.5 |  | 0.6 (0.4,0.8) |
| <= 39 | 0.0618 (0.0471, 0.0811) | 0.0723 (0.0583, 0.0897) | 18.0 | 0.679 | 1.0 (-1.4,3.4) |
| 40-49 | 0.0603 (0.0508, 0.0715) | 0.0662 (0.0574, 0.0764) | 10.0 | 0.636 | 0.8 (-0.7,2.4) |
| 50-59 | 0.0665 (0.0608, 0.0728) | 0.0726 (0.0668, 0.0788) | 9.1 | 0.778 | 0.5 (-0.3,1.4) |
| 60-69 | 0.0810 (0.0771, 0.0852) | 0.0829 (0.0788, 0.0872) | 1.2 | 0.515 | 0.2 (-0.3,0.7) |
| 70-79 | 0.0895 (0.0867, 0.0924) | 0.0944 (0.0914, 0.0974) | 5.6 | Ref | 0.4 (0.1,0.8) |
| 80-89 | 0.1059 (0.1031, 0.1088) | 0.1131 (0.1104, 0.1159) | 7.6 | 0.657 | 0.5 (0.2,0.8) |
| >= 90 | 0.1246 (0.1191, 0.1303) | 0.1461 (0.1411, 0.1513) | 17.7 | **0.004** | 1.2 (0.8,1.7) |
| Men | 0.0997 (0.0978, 0.1017) | 0.1059 (0.1040, 0.1079) | 6.1 | Ref | 0.5 (0.3,0.7) |
| Women | 0.0858 (0.0841, 0.0876) | 0.0921 (0.0902, 0.0940) | 8.2 | 0.609 | 0.6 (0.4,0.8) |
| Most affluent (Q5) | 0.0929 (0.0898, 0.0961) | 0.0976 (0.0947, 0.1006) | 5.4 | Ref | 0.6 (0.3,0.9) |
| Least affluent (Q1) | 0.0942 (0.0916, 0.0970) | 0.1022 (0.0994, 0.1051) | 8.5 | 0.555 | 0.5 (0.1,0.9) |
| White | 0.0945 (0.0929, 0.0961) | 0.1002 (0.0986, 0.1018) | 6.4 | Ref | 0.5 (0.3,0.7) |
| South Asian | 0.0911 (0.0838, 0.0989) | 0.0931 (0.0873, 0.0993) | 2.2 | 0.463 | 0.1 (-0.9,1.0) |
| Black | 0.0896 (0.0801, 0.1003) | 0.1104 (0.1019, 0.1196) | 23.6 | 0.156 | 1.3 (0.1,2.6) |
| Mixed/other | 0.0893 (0.0796, 0.1002) | 0.0957 (0.0881, 0.1039) | 6.7 | 0.832 | 0.6 (-0.7,1.9) |
| Unknown | 0.0874 (0.0847, 0.0902) | 0.0922 (0.0876, 0.0971) | 5.7 | 0.419 | 0.3 (-0.2,0.8) |
| Other CV | | | | | |
| All | 0.0736 (0.0723, 0.0749) | 0.0734 (0.0721, 0.0748) | 0.0 |  | 0.0 (-0.2,0.2) |
| <= 39 | 0.0629 (0.0492, 0.0806) | 0.0525 (0.0419, 0.0657) | -16.1 | 0.432 | -1.4 (-4.0,1.1) |
| 40-49 | 0.0570 (0.0486, 0.0669) | 0.0511 (0.0442, 0.0591) | -10.5 | 0.392 | -1.1 (-2.7,0.6) |
| 50-59 | 0.0518 (0.0472, 0.0568) | 0.0515 (0.0472, 0.0561) | 0.0 | 0.967 | -0.3 (-1.3,0.7) |
| 60-69 | 0.0631 (0.0600, 0.0665) | 0.0578 (0.0548, 0.0610) | -9.5 | 0.110 | -0.9 (-1.5,-0.3) |
| 70-79 | 0.0711 (0.0688, 0.0734) | 0.0683 (0.0661, 0.0707) | -4.2 | Ref | -0.3 (-0.6,0.1) |
| 80-89 | 0.0858 (0.0835, 0.0881) | 0.0855 (0.0834, 0.0877) | 0.0 | 0.403 | -0.1 (-0.4,0.2) |
| >= 90 | 0.1031 (0.0986, 0.1078) | 0.1196 (0.1156, 0.1238) | 15.5 | **<0.001** | 1.2 (0.7,1.6) |
| Men | 0.0770 (0.0752, 0.0787) | 0.0773 (0.0756, 0.0790) | 0.0 | Ref | 0.1 (-0.2,0.3) |
| Women | 0.0699 (0.0683, 0.0716) | 0.0687 (0.0671, 0.0704) | -1.4 | 0.436 | -0.1 (-0.3,0.2) |
| Most affluent (Q5) | 0.0742 (0.0715, 0.0770) | 0.0748 (0.0723, 0.0774) | 0.0 | Ref | -0.1 (-0.5,0.3) |
| Least affluent (Q1) | 0.0734 (0.0711, 0.0758) | 0.0719 (0.0696, 0.0743) | -2.7 | 0.633 | 0.1 (-0.4,0.5) |
| White | 0.0752 (0.0738, 0.0766) | 0.0752 (0.0739, 0.0766) | 0.0 | Ref | 0.1 (-0.1,0.3) |
| South Asian | 0.0581 (0.0524, 0.0645) | 0.0571 (0.0526, 0.0619) | -1.7 | 0.563 | -0.4 (-1.6,0.8) |
| Black | 0.0607 (0.0528, 0.0698) | 0.0514 (0.0458, 0.0576) | -15.0 | 0.096 | -1.4 (-3.0,0.3) |
| Mixed/other | 0.0676 (0.0592, 0.0773) | 0.0591 (0.0533, 0.0655) | -11.9 | 0.214 | -0.9 (-2.4,0.7) |
| Unknown | 0.0706 (0.0682, 0.0731) | 0.0708 (0.0668, 0.0751) | 0.0 | 0.591 | -0.1 (-0.6,0.5) |
| Non CVD | | | | | |
| All | 0.133 (0.131, 0.135) | 0.167 (0.165, 0.169) | 25.6 |  | 1.9 (1.8,2.0) |
| <= 39 | 0.111 (0.0954, 0.129) | 0.131 (0.1162, 0.148) | 18.0 | 0.456 | 1.3 (-0.5,3.1) |
| 40-49 | 0.100 (0.0906, 0.110) | 0.114 (0.1055, 0.124) | 14.0 | 0.06 | 0.8 (-0.4,2.0) |
| 50-59 | 0.098 (0.0928, 0.104) | 0.118 (0.1125, 0.124) | 20.4 | 0.203 | 1.5 (0.8,2.2) |
| 60-69 | 0.111 (0.1076, 0.115) | 0.136 (0.1323, 0.140) | 22.5 | 0.149 | 1.6 (1.2,2.1) |
| 70-79 | 0.127 (0.1240, 0.129) | 0.162 (0.1587, 0.165) | 27.6 | Ref | 2.0 (1.7,2.3) |
| 80-89 | 0.148 (0.1460, 0.151) | 0.188 (0.1856, 0.191) | 27.0 | 0.674 | 2.0 (1.7,2.2) |
| >= 90 | 0.175 (0.1697, 0.180) | 0.223 (0.2185, 0.228) | 27.4 | 0.916 | 2.0 (1.6,2.4) |
| Men | 0.137 (0.135, 0.139) | 0.171 (0.168, 0.173) | 24.8 | Ref | 1.8 (1.7,2.0) |
| Women | 0.129 (0.127, 0.131) | 0.163 (0.161, 0.166) | 26.4 | 0.261 | 2.0 (1.8,2.1) |
| Most affluent (Q5) | 0.128 (0.124, 0.131) | 0.160 (0.156, 0.163) | 25.0 | Ref | 2.0 (1.8,2.3) |
| Least affluent (Q1) | 0.141 (0.138, 0.144) | 0.180 (0.176, 0.184) | 27.7 | 0.275 | 1.8 (1.5,2.2) |
| White | 0.137 (0.135, 0.139) | 0.170 (0.168, 0.172) | 24.1 | Ref | 1.8 (1.7,1.9) |
| South Asian | 0.117 (0.109, 0.125) | 0.163 (0.155, 0.171) | 39.3 | **0.018** | 2.7 (1.9,3.5) |
| Black | 0.125 (0.114, 0.138) | 0.152 (0.142, 0.162) | 21.6 | 0.982 | 1.8 (0.7,2.9) |
| Mixed/other | 0.126 (0.114, 0.138) | 0.141 (0.132, 0.151) | 11.9 | 0.171 | 1.1 (0.0,2.2) |
| Unknown | 0.119 (0.116, 0.122) | 0.136 (0.130, 0.142) | 14.3 | **0.001** | 1.1 (0.7,1.6) |
| With the exception of age groups, all predictions are at the mean population age (79 years). All proportions are estimated using the sum of first readmissions or deaths within 30-days of discharge from hospital as the numerator and all live discharges as the denominator. Live discharges are from all survivors of the index admission with HF, during the study time window (1st January 2002 and 31st December 2018).  CV, cardiovascular; Relative diff., relative difference; CI, confidence interval.  a relative percentage difference in admission proportions between the first and second diagnosis time periods, calculated by 100*([time-period 2 – time period 1] / time-period 1].  b P value for the difference in trend lines between groups, compared to the reference group. Estimated by fitting an interaction term between calendar year and group in the quasipoisson models also containing age.  c Average annual percentage change in rates (per 100 person-years) for each increasing year of index HF admission | | | | | |

| S4 Table: Associations between patient and clinical characteristics and readmissions by cause | | | | |
| --- | --- | --- | --- | --- |
|  | Odds ratio (95% CI) | | | |
| Variable name | All-cause readmissions | Heart Failure | Other Cardiovasular | Non Cardiovascular |
| Male sex | 1.0 | 1.0 | 1.0 | 1.0 |
| Female sex | 0.96 (0.95,0.97) | 0.87 (0.85,0.89) | 1.03 (1.00,1.06) | NA (NA,NA) |
| Ethnicity: White | 1.0 | 1.0 | 1.0 | 1.0 |
| Ethnicity: South Asian | 1.01 (0.97,1.04) | 1.10 (1.04,1.16) | 0.94 (0.86,1.02) | 0.98 (0.93,1.02) |
| Ethnicity: Black | 1.03 (0.99,1.08) | 1.32 (1.23,1.42) | 0.86 (0.77,0.96) | 0.92 (0.87,0.98) |
| Ethnicity: Other/mixed | 1.01 (0.96,1.05) | 1.14 (1.05,1.23) | 1.07 (0.96,1.18) | 0.91 (0.85,0.96) |
| Ethnicity: Unknown | 0.79 (0.77,0.80) | 0.85 (0.82,0.89) | 0.84 (0.79,0.88) | 0.77 (0.75,0.79) |
| IMD quintile: 5 (least affluent) | 1.0 | 1.0 | 1.0 | 1.0 |
| IMD quintile: 4 | 1.01 (0.99,1.04) | 1.03 (0.99,1.07) | 0.99 (0.94,1.05) | 1.01 (0.98,1.03) |
| IMD quintile: 3 | 1.03 (1.01,1.05) | 1.01 (0.98,1.05) | 1.00 (0.95,1.06) | 1.04 (1.01,1.07) |
| IMD quintile: 2 | 1.08 (1.06,1.11) | 1.06 (1.02,1.10) | 1.03 (0.98,1.08) | 1.09 (1.06,1.12) |
| IMD quintile: 1 (most deprived) | 1.13 (1.10,1.15) | 1.07 (1.03,1.11) | 1.02 (0.97,1.07) | 1.16 (1.13,1.19) |
| IMD quintile: Unknown | 0.74 (0.69,0.79) | 0.67 (0.59,0.76) | 0.74 (0.63,0.88) | 0.82 (0.76,0.90) |
| Age: spline coefficient 0 | 1.0 | 1.0 | 1.0 | 1.0 |
| Age: spline coefficient 1 | 1.21 (1.16,1.27) | 1.27 (1.17,1.39) | 0.67 (0.60,0.74) | 1.40 (1.31,1.49) |
| Age: spline coefficient 2 | 0.62 (0.49,0.79) | 1.22 (0.80,1.86) | 0.23 (0.14,0.38) | 0.66 (0.49,0.90) |
| Age: spline coefficient 3 | 0.99 (0.84,1.17) | 0.98 (0.73,1.33) | 0.27 (0.17,0.41) | 1.32 (1.07,1.63) |
| Admission year | 1.01 (1.01,1.01) | NA (NA,NA) | 0.99 (0.99,0.99) | 1.02 (1.02,1.02) |
| Specialty: non cardiology | 1.0 | 1.0 | 1.0 | 1.0 |
| Specialty: cardiology | 0.92 (0.89,0.94) | 0.93 (0.90,0.97) | NA (NA,NA) | 0.89 (0.86,0.92) |
| Specialty: unknown | 0.98 (0.89,1.07) | 0.86 (0.73,1.02) | NA (NA,NA) | 1.09 (0.97,1.22) |
| AF | 0.96 (0.95,0.98) | 0.98 (0.96,1.00) | 1.03 (0.99,1.06) | 0.95 (0.93,0.96) |
| Anaemia | NA (NA,NA) | 0.91 (0.86,0.96) | 0.83 (0.76,0.90) | 1.09 (1.05,1.13) |
| Arthrosis | 0.98 (0.95,1.00) | 0.97 (0.92,1.01) | 0.84 (0.78,0.89) | NA (NA,NA) |
| Asthma | 1.06 (1.04,1.09) | NA (NA,NA) | 0.92 (0.86,0.98) | 1.13 (1.10,1.17) |
| Cancer | 1.28 (1.25,1.32) | 0.94 (0.89,0.99) | 0.90 (0.83,0.96) | 1.51 (1.47,1.56) |
| CKD | 1.26 (1.24,1.29) | 1.32 (1.29,1.36) | NA (NA,NA) | 1.23 (1.20,1.26) |
| Male sexCOPD | 1.30 (1.28,1.33) | NA (NA,NA) | 0.90 (0.86,0.94) | 1.53 (1.49,1.56) |
| CVA | 1.18 (1.12,1.24) | NA (NA,NA) | 1.19 (1.06,1.33) | 1.25 (1.18,1.33) |
| Dementia | 1.17 (1.13,1.21) | 0.87 (0.82,0.93) | 0.85 (0.78,0.93) | 1.36 (1.31,1.41) |
| Depression | 1.19 (1.15,1.24) | NA (NA,NA) | 0.88 (0.79,0.97) | 1.30 (1.24,1.36) |
| Diabetes | 1.14 (1.12,1.15) | 1.15 (1.12,1.18) | NA (NA,NA) | 1.14 (1.11,1.16) |
| Hypertension | 0.93 (0.91,0.94) | 0.92 (0.90,0.94) | NA (NA,NA) | 0.93 (0.91,0.94) |
| Rheumatoid arthritis | 1.16 (1.11,1.22) | NA (NA,NA) | NA (NA,NA) | 1.23 (1.17,1.30) |
| CABG | 1.33 (1.18,1.50) | NA (NA,NA) | 1.23 (0.95,1.59) | 1.55 (1.35,1.79) |
| ICD | 0.69 (0.56,0.84) | 0.50 (0.33,0.76) | NA (NA,NA) | 0.82 (0.64,1.05) |
| CRT | 0.90 (0.80,1.01) | 0.59 (0.46,0.76) | NA (NA,NA) | NA (NA,NA) |
| PCI | NA (NA,NA) | 0.74 (0.61,0.90) | 1.49 (1.23,1.79) | NA (NA,NA) |
| Pacemaker | NA (NA,NA) | 0.69 (0.60,0.80) | 1.23 (1.05,1.44) | NA (NA,NA) |
| CI, Confidence Interval; AF, Atrial fibrillation; COPD, Chronic obstructive pulmonary disease; CRT, Cardiac resynchronisation therapy; CVA, Cerebrovascular accident; ICD, Implantable cardioverter defibrillator; IMD, Index of Multiple Deprivation; PCI, Percutaneous coronary intervention  Note: NA indicates that the variable was removed from the model in the backwards AIC process | | | | |

# **S1 Figure: Cohort selection flowchart**

**698,983** index admissions

**699,030** index admissions

**699,062** index admissions

**711,061** index admissions

**1,069,431** All emergency admissions for heart failure (HF) within the period 1^st^ Jan 2001 to 31^st^ Dec 2018, for patients aged between 18 and 120

**357,544** excluded for not being the first emergency HF admission within the time period (2001-2018), or for being before 1^st^ Jan 2002

**826** excluded for being “duplicate” spells – same patient and admission date meaning not possible to identify index admission.

318,498 non-elective operations excluded

**32** excluded because readmission date after date of death

**11,999** excluded because final discharge date (end of super-spell) could not be confidently identified

**47** excluded because of unknown sex

# **S2 Figure: Effect of age on probability of readmission**

All readmission Heart failure


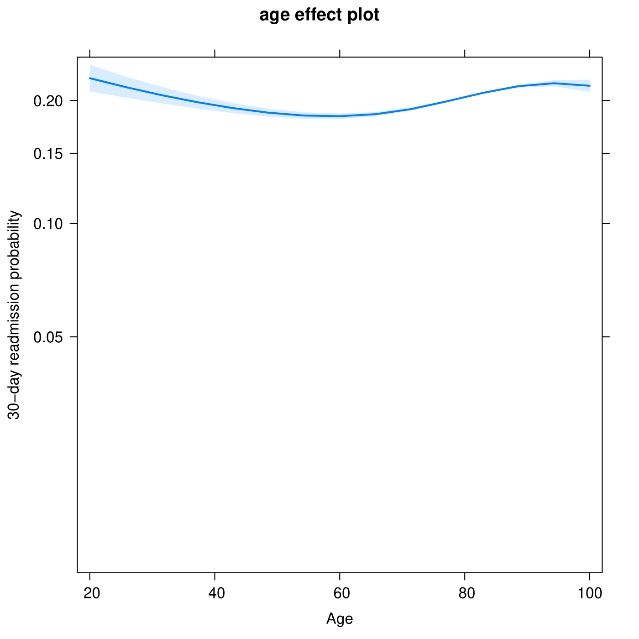

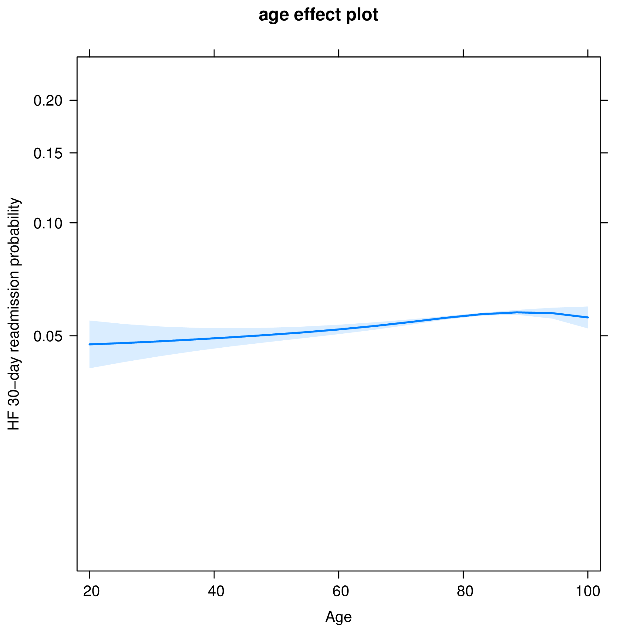


Other CVD Non-CVD


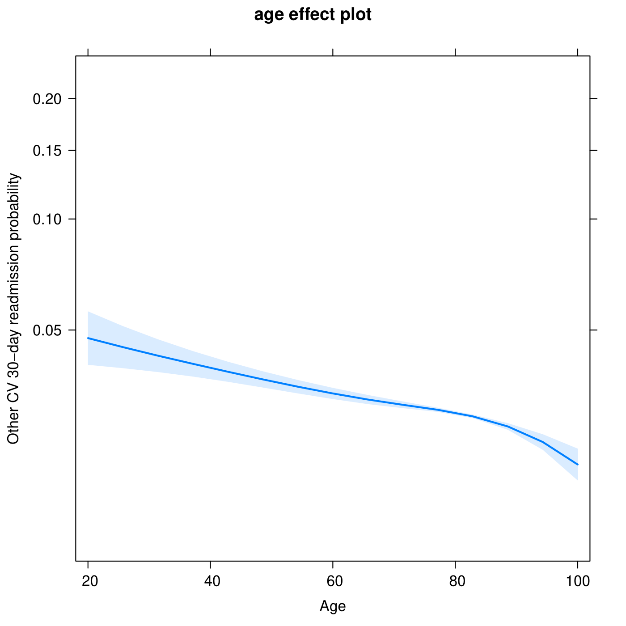

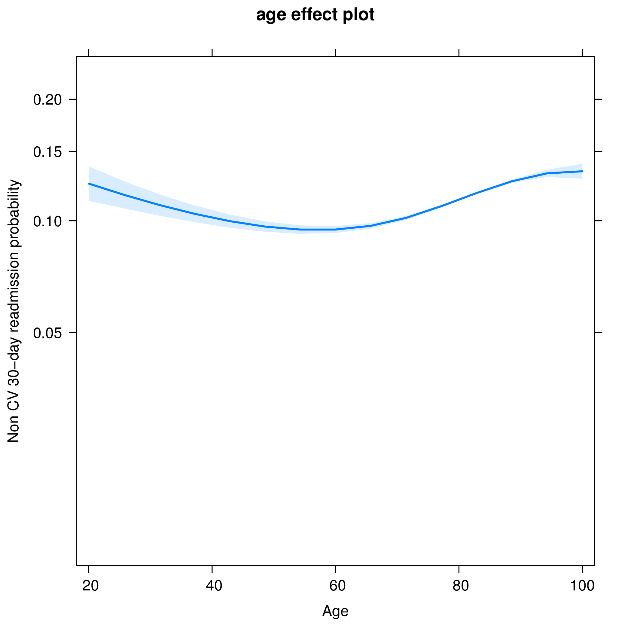


Age was entered as a cubic spline with 3 knots into the quasi-poisson models containing all covariates. The graphs show the predicted probability of readmission for different ages if all of the other variables are held at their mean values.
